# The poetics of vulnerability: creative writing among young adults in treatment for psychosis in light of Ricoeur’s and Kristeva’s philosophy of language and subjectivity

**DOI:** 10.1007/s11019-020-09998-5

**Published:** 2021-01-16

**Authors:** Oddgeir Synnes, Kristin Lie Romm, Hilde Bondevik

**Affiliations:** 1 Centre of Diaconia and Professional Practice, VID Specialized University, Oslo, Norway; 2 Institute of Clinical Medicine, Norwegian Centre for Mental Disorders Research, Faculty of Medicine, University of Oslo, Oslo, Norway; 3 Division of Mental Health and Addiction, Oslo University Hospital, Oslo, Norway; 4 Department of Interdisciplinary Health Sciences, University of Oslo, Oslo, Norway

**Keywords:** Creative writing, Psychosis, Young adults, Paul Ricoeur, Julia Kristeva

## Abstract

There is a growing interest in the application of creative writing in the treatment of mental illness. Nonpharmacological approaches have shown that access to poetic, creative language can allow for the verbalisation of illness experiences, as well as for self-expressions that can include other facets of the subject outside of the disease. In particular, creative writing in a safe group context has proven to be of particular importance. In this article, we present a pilot on a creative writing group for young adults in treatment for psychosis. We set the texts and experiences from the writing group in dialogue with Paul Ricoeur’s and Julia Kristeva’s philosophies on *poetic language* as meaning making and part of subject formation. The focus is on language as materiality and potentiality and on the patient’s inherent linguistic resources as founded in a group dynamic. As a whole, the project seeks to give an increased theoretical and empirical understanding of the potentiality of language and creativity for healing experiences, participation and meaning-making processes among vulnerable people. Furthermore, a practice founded in poetic language might critically address both the general and biomedical understanding of the subject and disease.

## Introduction

‘For all miracles are powerless to prevent the expression of ideas in writing; the occasional attempt to paralyze my fingers, though making writing somewhat difficult, does not prevent it, and attempts at disturbing my thoughts are easily overcome by putting them down in writing during which one has a great deal of time to collect one’s thoughts’.

Daniel Paul Schreber 1903

At the beginning of the last century, the distinguished judge Daniel Paul Schreber used writing to make sense of and tell his story. He suffered from delusions and voice hearing and felt mentally and physically ‘tortured’ by what he called ‘miracles’. Even so, he was able to write one of the world’s most famous books about psychotic experiences to regain his liberty from the asylum where he was confined. Beyond a doubt, Schreber used writing and music as a means to bring order and peace to his mind. He developed a belief in his own powers by being able to confer important matters to the outside world.

Over the past few decades, there has been growing interest in creative arts therapy and other psychotherapies in the treatment of mental illness. Music, visual art and theatre have moved from an activity of meaningful occupation of time to being recognised as a therapeutic tool. Art therapy is mostly used as an adjunct to medication, but user organisations have also advocated for more alternatives when adverse effects or a lack of effect makes medication intolerable (Hanevik et al. 2013). Creative and expressive writing includes personal journaling, poetry, fiction and autobiographic memoirs, and this form of writing has been acknowledged as a therapeutic tool for people suffering from severe mental illness (Chiang et al. 2019; King et al. 2013; Stuckey and Nobel 2010). Access to poetic, creative language may allow for the verbalisation of illness experiences, as well as self-expressions that can include other facets of the subject that rest outside of the disease. Creative writing in a safe group context allows for peer-to-peer support, and this might be especially important for people with severe mental health challenges. Even though it could be provided as individual therapy, group sessions have been recommended to optimise outcomes (Bundesen and Rosenbaum 2020; Hanevik et al. 2013).

In the current paper, we present a pilot project on a creative writing group for young adults with psychosis; here, we look into written texts and excerpts from interviews with the participants. To illuminate the potential of creative writing in this group, we analyse the project in light of two philosophers’ work on poetic language. Paul Ricoeur’s long-standing work on *poetics of being* (1950, 2008b) underscores how poetic language is central for providing new understandings of ourselves as capable beings in the world. According to Ricoeur, our subjectivity depends on language and symbols; thus, self-understanding requires interpretation through available linguistic resources. Additionally, an important premise for our work is Julia Kristeva’s (1974) dynamic understanding of the subject and the idea that treatment cannot be approached by looking at patients ‘as objects under treatment’ but only as ‘emerging subjects’. Her understanding of the ‘semiotic’ as fundamental for meaning making and symbolisation is discussed in relation to the selected texts. As a whole, the project seeks to give an increased theoretical, as well as empirical, understanding of the potentiality of language and creativity for healing experiences, participation and meaning-making processes among vulnerable people, as well as to critically address the biomedical understanding of the subject and disease.

More specifically, in light of Ricoeur’s and Kristeva’s perspectives, the aim of the article is to investigate the following: *How can poetic language be a resource for young adults in treatment for psychosis through participation in a safe and supportive creative writing group?*

## Psychosis among young adults and the need for nonpharmacological interventions

Being psychotic is one of the most distressing and debilitating conditions a person can experience. A psychotic episode is often characterised by symptoms such as hallucinations, thought disorder, delusions or change in feelings and emotions, and it is followed by both intrapersonal and interpersonal distress (Griffiths et al. 2019). Psychotic disorders often start in late adolescents/early adulthood. This is an age marked by a demand for increased independence, acceptance of friends, separation from parents and where school results are a yardstick for success. A person who develops psychosis will often experience difficulties in several of these areas, and loneliness among this population is common (Lim et al. 2018). They may feel more dependent on their parents again, feel alienated from their friends, feel stigma and shame and may experience cognitive difficulties related to the disorder. The high degree of misinformation about the prognosis associated with these conditions, especially with schizophrenia, tends to underestimate the chances for recovery and adds to the burden. It is easy to lose hope even though the vast majority will either recover or be able to live meaningful lives, even with some symptoms persisting (Lally et al. 2017).

However, since the 1990s, there has been a move towards a more recovery-oriented approach towards treatment, where the importance of personal resources and meaning has been emphasised (Chester et al. 2016; Lysaker et al. 2018; Slade et al. 2014). Recovery is not only about reducing psychotic symptoms: the ability to experience connectedness, hope, identity, meaning of life and empowerment has been highlighted as well (Slade et al. 2014). Furthermore, there has been an increased focus on personal recovery as opposed to clinical recovery: ‘the development of new meaning and purpose in one’s life, as one grows beyond the catastrophic effects of mental illness’ versus remission in symptoms and functional improvement (Anthony 1993; Van Eck et al. 2017). Acquiring hope and self-confidence and overcoming symptoms and stigma through mobilising resources are important for personal recovery.

To promote recovery, the use of strength-based interventions has been highlighted. Previous research has defined 24 character strengths, among them creativity, persistence, social intelligence and hope (Linley et al. 2007). Creative writing bridges into art therapy, where creative activity has been found to improve mental health, satisfaction with care, social and cognitive functioning and overall well-being in different patient populations, even though the results of its effectiveness have been mixed (Chiang et al. 2019). The varied outcomes may be attributed to a range of reasons, but high-quality qualitative papers indicate that both therapists and clients find it meaningful, beneficial and acceptable (Attard and Larking 2016).

## Previous research on creative writing in (mental) health care

Research on writing among different patient groups has been diverse in terms of these studies’ methodologies. One direction that has received much attention is James Pennebaker’s research on *expressive writing*, where over several individual writing sessions various groups of patients have been asked to write about traumatic life events for 20 to 30 min. Expressive writing is a nonliterary intervention, with no emphasis on genre or literary techniques. The vast amount of research on expressive writing has shown various positive effects through, for example, fewer doctor visits, reduced self-reported illness and better management of the disease (Pennebaker 1997, 2000, 2010).

Although *expressive writing* is a clearly defined method that has been researched extensively, *creative writing* is a much less defined practice, covering various forms of literary writing such as poetry, fiction and storytelling, both in groups and on an individual basis (Costa and Abreu 2018; Gillam 2018). Costa and Abreu (2018) call for greater clarity with a consistent conceptualisation for the application of creative writing in clinical settings; they conclude that at the moment, there are no ‘established ways of assessing qualitatively or quantitatively the therapeutic benefits of creative writing’ (2018, p. 83).

One tradition under the umbrella of creative writing is poetry therapy, which is defined as ‘the use of the written or spoken word to further therapeutic goals and enhance the well-being of individuals, families, couples, or groups […] poetry therapy promotes growth and healing through expressive writing activities and through the reading and facilitated discussion of literary material’ (National Association for Poetry Therapy n.d.). The benefits of poetry therapy involve increased self- and interpersonal awareness, validation of voice and increased capacity for capturing and redescribing the significant life events. According to Kenneth Gorelick, a seminal figure in poetry therapy, the goal of poetry therapy is to provide opportunities to describe loss, but also to find a language that can fulfil hope (2005). This short presentation of poetry therapy is a characteristic example of the open-ended nature of the various practices that are in use. This understanding of poetry therapy could easily be applied to several other practices lumped under the umbrella of creative writing. Another example is McArdle and Byrt’s (2001) definition of expressive writing in mental health as ‘the use of writing to enable people with mental health problems to enjoy and express themselves, develop creativity and empowerment, affirm identity and give voice to views and experience’ (p. 517). Even though the latter use the term ‘expressive writing’, it is clear that they differ from Pennebaker’s highly structured and individual approach and are more related to how poetry therapy presents itself. The lack of a homogeneous practice in creative writing makes it difficult to compare outcomes across studies (Costa and Abreu 2018).

In recent years, mental health care has seen an increased interest in art therapeutic directions, such as visual arts (Crawford et al. 2012; Green 1987), music (Yang et al. 1998; Ulrich et al. 2007) and variants of creative writing (Houlding and Holland 1988; McArdle and Byrt 2001; Shafi 2010). However, there are only a few studies on the use of creative writing in severe mental illness. Houlding and Holland (1988) report that participants in a creative writing group among people with severe mental difficulties found that group affiliation allowed for greater contact and less isolation. In addition, writing provided opportunities to express their overwhelming emotions. This has been supported by McAardle and Byrt (2001) and Shafi (2010), who argue that creative writing can help express oneself and control thoughts and hallucinations. Several case reports also describe how writing can be markers on the road to recovery (Hankir et al. 2012; Kar and Barreto 2018). These findings are in line with the preliminary results from the Danish research programme ‘Rewritalize—creative writing groups as part of psychiatric treatment’, which is an interdisciplinary project that has applied a mixed method design and consists of one art practice part and one research part (Bundesen and Rosenbaum 2020). Their findings highlight how creative writing in a group setting in psychiatric treatment can have several positive psychological effects, such as symbolic meaning making, mentalising and the enhancement of interpersonal abilities.

Some studies have explored frameworks that propose theoretical links between creative writing and recovery from severe mental illness. King et al. (2013) outline three different theoretical perspectives to investigate frameworks for creative writing as part of a recovery process: (1) the relationship between the narrative and the emergence of identity, (2) writing as a means to reconstitute a void in the internal symbolic order of the person and (3) creative writing as a form of cognitive remediation. These perspectives might be especially valuable for people who suffer from psychosis with self-disturbances and identity problems (Sass et al. 2018) and/or cognitive impairment (Karr and Singh 2019). Both self-disturbance, which is a phenomenon of disruption or diminishing of a person’s sense of minimal (or basic) self, and cognitive impairment are considered to be major challenges (Sheffield et al. 2018). A related approach comes from Bundesen and Rosenbaum (2020), who combine theoretical perspectives from psychoanalysis, literary theory, and phenomenology to explore creative writing’s potential healing processes.

An interesting additional aspect that is argued for by King et al. (2013) and Bundesen and Rosenbaum (2020) is the importance of focusing on *literary writing techniques* for therapeutic value, emphasising both the importance of learning from a writer, not a health professional, and writing as a skill for expressing oneself.

The current project was designed as an exploratory study where we wanted to gain more insights into the processes and experiences of a group with young adults living with psychosis that were given the opportunity to participate in the writing of creative texts in a safe and supportive group environment.

## Theoretical perspectives

The theoretical perspectives applied in the current article are based on two philosophers who argue for the centrality of poetic language in the formation of human experiences and subjectivity. For both Ricoeur and Kristeva, poetic language is a vital resource for agency, self-understanding and self-experience. Through poetic language and linguistic repertoires in culture, the self can find new ways of being. We argue that a look at the aspects of Ricoeur’s and Kristeva’s theories will give an increased understanding of what can be at stake when working with creative writing among vulnerable patients.

### Paul Ricoeur and the poetics of human existence

Throughout his philosophy, Paul Ricoeur has been concerned with how human existence always is situated between the voluntary and involuntary, between nature and culture and between the given and the chosen: ‘Living is already having been born, in a condition we have not chosen, a situation in which we find ourselves, a quarter of the universe in which we may feel we have been thrown and are wandering, lost. And yet it is against this background that we can begin, that is to say, give a new course to things …’ (Ricoeur 2008a, p. 211). As human beings, we are part of nature and vulnerable to diseases, to death and to other people’s actions, while at the same time, we have a capability conditioned by the abilities of a body situated in a particular context. For Ricoeur, human existence is given in language and conditioned by the culture we are a part of. We can never have direct access to ourselves; the self can only be seized indirectly through an interpretation of itself through the available linguistic and symbolic resources found in one’s culture: myths, stories, different beliefs and discursive repertoires.

Although the linguistic repertoire of a culture can always be interpreted as limiting, Ricoeur wants to show how ‘human language is *inventive* despite the objective limits and codes which govern it…’ (Ricoeur, in Valdés 1991b, p. 465). In particular, Ricoeur has been occupied with how poetic language as *poiesis*, as productive work, opens up possibilities for how we understand ourselves in the world.^1^ This perspective is apparent in the works of Ricoeur that investigate what he terms *semantic innovation*, where the creative language tools of *metaphor* (2008b) and *narrative* (1992) are central for how we come to orient ourselves in the world through an indirect hermeneutics, by interpretation on the level of the sentence (metaphor) and through the plot (narrative).

For Ricoeur, literature and poetic language demonstrate man’s capability in the way it ‘preserves the width, the breadth of language’ (Ricoeur, in Valdés 1991a, p. 448) by its plurivocity. As such, it is the opposite of the language of science and technology, which aims at an instrumentalisation of language as a striving for language and reality to be one. Poetic language, on the other hand, implies potentiality and possibilities, not as correspondence but as a redescription of the world: ‘Is it not the function of poetry to establish another world—another world that corresponds to other possibilities of existence, to possibilities that would be most deeply our own?’ (Ricoeur 2008b, p. 270–271). As such, Ricoeur sees poetic images closer to a verb than a portrait, something that opens the world to a potential ‘as if’. Poetic images as verbs thus ‘reveal our being-in the-world while […] being also “our work” as a manifestation of human possibilities’ (Helenius 2013, p. 63). Poetic images are thereby productive for human potentiality and understanding, or in Ricoeur’s words, can open for *a poetics of being* (*une ‘poétique’ de l’être*) (1950).

An important factor in how poetic language opens up such perspectives of potentialities is what Ricoeur terms the ‘hermeneutical function of distanciation’ of the text (1981). When a discourse becomes a text, it frees itself from the writer and the author’s intention, opening itself up for infinite readings and possibilities for the reader: ‘For what must be interpreted in a text is a *proposed world* which I could inhabit and wherein I could project one of my ownmost possibilities’ (1981, p. 142). However, this proposed world that the poetic discourse opens up for is not something to be found as a hidden intention *behind* the text but is found in front of it through the act of the subject’s interpretation: ‘to understand is *to understand oneself in front of the text* […] of exposing ourselves to the text and receiving from it an enlarged self…’ (1981, p. 143). Thus, Ricoeur’s understanding of the subject is that it is always in the process of becoming through the mediating encounters of the texts in the culture. It is a creative interpretive act that might open up new realities and possibilities for the self.

Ricoeur’s philosophy provides a perspective on the prerequisites for meaning making that can involve a liberating creative process, one that may potentially be important for persons with a severe psychiatric disorder. For instance, one important point for Ricoeur is how the confrontation between the individual and different discourses can enable or hinder language and action. In the extension of this, the literary language through poetry and narrative becomes central for a potential therapeutic function in that this becomes a language that can imply a cleansing (katharsis) within the practice of working through—narrative and literature have the potential to reinterpret one’s life. Although one is never the author of one’s existence, one can be an author of its meaning, as Ricoeur writes (1992). On the other hand, poetic language can also imply a way for the wounded and marginalised to find a voice, as well as a release of human potential: ‘…through this recovery of the capacity of language to create and re-create, we discover reality itself in the process of being created […] Language in the making celebrates reality in the making’ (Ricoeur, in Valdés 1991a, p. 462).

Another aspect of Ricoeur’s philosophy that might be of particular relevance for this specific writing group of young adults with psychosis is the emphasis of understanding as something that happens *in front of the text*. An important part of the writing group is the freedom attached to the knowledge that it is the meaning of the text, not necessarily the author’s intention, that is in focus. This is one advantage of a poetic discourse; it involves possible worlds that do not necessarily reveal the inner world of the writer. In Ricoeur’s argument, poetic language opens this perspective in a double movement: ‘…one way of revealing is also one way of making more obscure’ (Ricoeur, in Valdés 1991a, p. 460). We believe this is of the utmost importance when we are working with creative writing among vulnerable patients. Poetic language can open up possible worlds for both the writer and reader.

### Julia Kristeva’s theory of language

The French psychoanalyst and philosopher Julia Kristeva, best known for her linguistic writings and her dynamic understanding of the subject, has throughout her career had a special interest in combining psychoanalytic theory with language and literature. In our study, we are especially inspired by Kristeva’s understanding of the ‘semiotic’ as fundamental for meaning making and symbolisation and her exploration of the possibilities that exist in the poetic language related to mental illness and psychosis.

According to Kristeva, every theory of language depends on a specific understanding of the subject. As she understands it, the subject is permanently ‘in process’ [subject-en-procès], always in a process of becoming; by nature, it is in motion (‘From One Identity to Another’) (1984b). In *La Révolution du langage poétique* (*Revolution in Poetic Language*) (1974), which was her doctoral thesis and also was her breakthrough, Kristeva describes that language appears as a polyphonic mosaic of transformed quotes in an intertextuality where the language is social and takes the form of dialogue (Bakhtin) as a result of the interaction between the individual and the environment. Language is dialogical also in the sense of being a divided signifying process, a production of meaning that seems to be an interaction between what Kristeva calls the *semiotic* and *symbolic*. Language itself consists of these two irreducible elements; they are linked together and could not be seen as dichotomous contradictions. With these concepts or processes, Kristeva also links the soul and body, the psyche and body and nature and culture in a dynamic way.

As speaking and writing subjects, we are also bodily beings whose bodily desires and energies—the *semiotic*—are in opposition in some way to the linguistic order—the *symbolic* (here, the semiotic as a kind of parallel to Freud’s unconscious). The semiotic, for its part, is further associated with rhythms, tones and movements, while the symbolic is associated with grammar and structure.

In this context, ‘normality’ is a sort of balancing act between the *semiotic* and *symbolic,* between the bodily and the linguistic, understood as the order, while ‘pathology’ may be seen as a loss of balance resulting in a fall to one side or another in this signifying process, either completely into the *semiotic* (and meaningless) or into the *symbolic* (and strictly legal) (1974).

Kristeva has also addressed several symptom dimensions and afflictions in her writings, such as depression, melancholy and hysteria, any of which may include psychotic elements. In *Revolution in Poetic Language* and later in *Black Sun—Depression and Melancholy* (1989), she looks at what it is in art and literature that may heal mental illness and distress.

As Kristeva understands it, depression is thought to be a form of denial of linguistic meaning, loss of meaning, loss of connections and relationships, loss of interest in the environment and, ultimately, loss of contact with bodily reality. This is where *poetic language*, according to Kristeva, *as one signifying practice*, can act as a reactivation of the semiotic, thus helping counteract an oppressive symbolic order, as well as strengthening the constructive dimensions in the language of the dissolving and destructive in a one-sided semiotic process.

Depression is anything but creativity. It is a loss of language and affiliation. However, the insanity, despair and turmoil—when strengthened by the semiotic—can be like dark matter that stimulates creativity. The melancholic temperament’s experiences of loss and absence can create the need to restore meaning and find a balanced language for these experiences to progress and move forward.

In our context, it is also interesting to note that in recent years, Kristeva, together with psychiatrist and Professor Marie Rose Moro, have explored the pathological and healing powers of cultures more concretely in the seminar on ‘Need to Believe’ (Kristeva 2011). This seminar is aimed at various professionals in the health sector who deal with cross-culture discontent among adolescents (Kristeva et al. 2018). Likewise, in ‘Interpréter le mal radical’ (2016), Kristeva refers to the case of Souad, a teenage girl from a Muslim family who suffered from severe anorexia and, subsequently, became more radicalised. Through psychotherapy with a multicultural team and writing and theatre workshop, including reading Arabic poetry translated into French, Souad gradually started to re-establish ties to the world and to her own body (Kristeva et al. 2018).

Taking Kristeva’s theory of the *subject* and *signification,* here understood as a production of meaning related to poetic language, we ask: Can Kristeva’s early writing on the semiotic and symbolic, and the concept of the polyphonic (*polyloge*), the diversity of rationality and of language contribute to our understanding of how language might maintain and strenghten life-giving and positive aspects of existence?

Further, is it outside of the institutionalised dialogue that the healing therapeutic potentials exist, opportunities for the ill subject to enable some of their intrinsic linguistic and bodily resources to create new meaning and affiliation, new life-giving connections and greater interest in the surroundings related to the subject?

Held together, the theories of Ricoeur and Kristeva bring a ‘poetic ethics’ or an ‘ethics of linguistics’ (1984a) to our project: by stressing how the subject is relational and discursively constituted, this highlights the need for an awareness of the potentials that creative language might bring the individual, both as a writer and as a reader/listener.

## Methodology

### The group

The participants were recruited from a department for in- and out-patient treatment for early psychosis at Oslo University Hospital. All participants had experienced periods with psychotic symptoms. The department is designed to provide a coherent treatment offer for a maximum of five years after a first episode of psychosis for people aged 18–30 years. When entering the service, most patients experience a moderate or high level of impairment because of psychotic symptoms. The project aimed to recruit people who were interested in writing in a natural setting, and it was stressed that this was an offer on the side and not to be counted as a part of their treatment; our programme did not interfere with their treatment as usual. We did not collect demographic data or data from hospital records and stressed that we were not interested in that part of their story unless they had something they wanted to share with the group. We held an open door, and seven persons participated during the course, while five participants became a core group and followed the course on a more permanent basis. These five produced the material for the present paper, and we have renamed them in the following way: three men (David, Gordon, Nick) and two women (Cindy and Joni).

### The writing practice

The group met once a week for 2 h each time. Initially, six to eight meetings were planned, but the course was expanded to 12 meetings after requests from the participants. The project took place in the hospital area but not in the department responsible for their treatment. Here, we aimed to use a more neutral space.

The group was facilitated by the three authors of the present article; the first and last author led the group activities, while the second author was there as a participating observer and as a health professional if a difficult situation related to their psychological well-being should arise during the sessions. The first author is a literary scholar working in health humanities who has worked with creative writing for many years in different health settings, such as cancer care, dementia care and palliative care. The second author is a psychiatrist with extensive experience in working with young adults and psychosis. She was also the link to the participants’ therapists and worked at the setting where the group took place. The last author is a scholar in medical humanities with an extensive experience on research on literary representations of illness.

The protocol was influenced by the philosophies of Kristeva and Ricoeur together with traditions in creative writing that emphasise fantasy, perspective taking and literary techniques, both from outside of clinical contexts (Goldberg 2005; Koch 1999) and inside of clinical contexts (Herman 2017) and in illness and loss (Gubar 2016; Orr 2002). Thus, the project was founded on theory and practices, as well as our own background from previous work that underscore creative writing as a potential for (self)discovery, joy, therapy and relation building.

Being a particularly vulnerable group, the group leaders spent significant time outlining the pedagogy of the group: this was meant to be a creative and supportive community where the emphasis would always be on what the participants achieved. By reading published poetry and prose, as well as reading the products of the participants, the group session would be devoted to looking for the potential and linguistic creativity displayed in the various texts, looking for metaphors, details, original expressions, contrasts and so forth. Thus, the learning of literary techniques was located in the participants’ own texts, as well as in other relevant literature that was read during sessions. The method of learning from each other’s texts, as well as from what the participants achieved, was pinpointed as especially important during the recruitment phase and the initial sessions. We also saw the need for repeating the method regularly during the sessions to lessen the fear of failure among some participants. Another important methodological aspect of the group was the highlighting of the difference between the author and their texts. Following Ricoeur, we stressed how texts are always linguistic constructions that do not necessarily mirror the participants’ inner self as correspondence. In the groups, the focus was to be on the text and the textual self, not on the author’s life, situation or intentions.

The first meeting consisted of an outline of the method and a presentation round of the group leaders and participants. We then read a poem and had a short conversation of its content and the way the poem was written. It was important that the participants experienced writing and mastering right from the start. The first writing exercise was based on a reading of a few opening paragraphs of Charles Bukowski’s novel *Ham on Rye,* which is a depiction of a childhood memory with visual details seen from a young boy’s perspective. We then asked the participants to write for 10 min, taking a good childhood memory as a starting point and focusing on the sensual details of the place they were and what happened there. Again, we stressed the importance of the process, of play and of trying to write with no pressure on the end result. The participants were asked to read aloud their texts, and the two group leaders commented on each text, underlining what we deemed as literary qualities and linguistic details in the texts. Through this first reading, the participants got to know the pedagogical method: we as leaders would comment on the various texts but never criticise them. We would comment on what we found interesting, what we liked and, in particular, highlight various ‘literary’ techniques that were (un)consciously employed in the texts, thereby encouraging and developing the participants’ attention towards their own texts and the reading of each other texts. The participants could also comment on each other but never in a critical way. After the round, we read a poem by the Norwegian poet Helge Torvund. We ended the session by giving them ‘homework’. They were asked to use the word ‘trust’, which was one of the words in the poem of Torvund, as a prompt and write something for the next session. We stressed that ‘homework’ was said humorously/tongue-in-cheek and that it was an opportunity, not an obligation, and that they also could write in response to other things than to this prompt too and in whichever genre they preferred.

At the very end, we had a short feedback session where the participants commented on how they had experienced being part of the group. The group session lasted approximately 2 h.

Each group meeting had a similar structure. We started by recapturing the last session, emphasising the literary technique or aspect that was in focus, before reading a poem or a short literary text and talking about the content and literary qualities. We then had a 10 min writing exercise dedicated to a literary technique, genre or a specific theme followed by a round of reading with comments. After a short break, we then read the texts that (some of) the participants had written as a home exercise, and we always ended with feedback on the session.

The highlights of the sessions were the writing exercises and the following discussions about the texts. In addition to writing using the senses, the writing tasks during the sessions consisted of various exercises that emphasised, for example, ‘fantasy’ (writing about yourself at an age that is decided by the roll of two dices), creating suspense by writing flash fiction of six words, writing dialogues, making abstract feelings specific through the use of certain metaphors and similes, writing from different perspectives or writing in different literary genres like short stories and poems (including haiku). The tasks in between meetings were always specific but open-ended writing prompts where the participants could decide for themselves what and how they wanted to write. Examples were ‘trust’, ‘what we are searching for’, ‘my heart’, ‘standing in the rain’, ‘what I would not change in my life’ and ‘write from the perspective of a dog, a tree or the universe’. At the last meeting, the participants wrote meta-texts about writing.

### Empirical material

The writing group resulted in around 100 texts consisting of poems, narrational fragments and some short stories. In addition, the three group leaders wrote field notes after the group sessions. Some weeks after the group had finished, qualitative interviews were conducted with the five participants. The interviews lasted between 15 and 48 min. All interviews were tape recorded and transcribed verbatim.

### Analysis

The analysis does not aim to give a representative presentation of the material as a whole but rather a reading of some significant texts that can illuminate particular aspects of how poetic language can be a resource. In the analysis, we have been inspired by Maggie MacLure’s concept of ‘wonder’ in qualitative research: ‘During the process of coding, some things gradually grow, or glow, into greater significance than others, and become the preoccupations around which thought and writing cluster’ (MacLure 2013a, p. 175). Furthermore, she writes how these things, be they, for example, a fragment, an anecdote or facial expression ‘…exert a kind of fascination, and have a capacity to animate further thought’ (2013b, p. 228). The analysis consisted of all three authors doing a close reading of the different texts that were produced during the groups, looking for the poetic qualities in each one, as well as the contents. What guided our reading was precisely looking for *wonder*, of texts that either said something original or profound about the resources of poetic language or creative writing or texts that had become important in the reading and reception in the group setting. In addition to analysing the texts, the impact of the poems for the authors in the poetry group was analysed in light of the field notes from these group settings and the semistructured interviews with the participants. Hence, the interviews were primarily used as supportive data to enrich the analysis of the texts and their potential meanings. The analysis is supplemented by the theoretical perspective in the discussion.

### Ethics

The project was evaluated by the Regional Ethical Committee (REK), who found it to be outside their scope, and it was approved by the data protection officer at Oslo University Hospital. Only people who were able to give written informed consent were invited to participate.

In the case of symptom exacerbation, we were in contact with each participant’s clinician and gave weekly feedback on how the course was moving along, and KLR followed up any signs of emotional strain that seemed to occur during sessions, both during sessions and in consultation afterwards, when needed.

There seems to be little risk associated with creative writing, and this is explained by the decisive emphasis on the participant’s freedom to decide what to write for the open assignments. However, a negative element is highlighted by Gallagher and Cole (2011), who show that writing in different genres for some patients with psychosis problems caused them to be less able to distinguish between reality and imagination.

### Findings: poetic practice as relatedness

We will answer the research question, *‘How can poetic language be a resource for young adults in treatment for psychosis through participation in a safe and supportive creative writing group?’* by presenting a few emblematic texts from the writing group supported by glimpses and snapshots from the group sessions, as well as from interviews with the participants.

Because the texts are grounded in poetic and productive language, the texts are naturally more or less open ended, lending themselves to different interpretations. Here, we are attempting to present one possible reading among several, emphasising poetic language as a *resource*. We do this by presenting texts under four headings that are grounded in our close reading of the material: (1) *writing as playful and reflective (co-)creation,* (2) *writing as a ‘long table’,* (3) *writing as individual discovery* and (4*) writing as mystery.*

### Writing as playful and reflective (co-)creation

‘Writing as playful and reflective (co-)creation’ highlights how the writing group became a room for discovery, sharing and play among the participants in the production and sharing of the texts. A fitting example is the group poems that were made at one of the meetings when we worked on making abstract feelings concrete through metaphor and similes by asking questions such as ‘what kind of landscape/sound/animal can represent the feeling of, for example, happiness, anger, fear.’ The participants worked spontaneously on visualising feelings by suggesting metaphors that were written on a flip chart. The metaphors suggested by the participants became group poems of various feelings, read aloud on the spot right after the last metaphor was added. The following poem was produced within a few minutes from responses from the participants regarding the feeling ‘unrest’:

**Unrest**

Unrest is

A rabbit

A beaver that hides

Unrest is

A desert

A quiet room

A kitchen knife

Both sharp and blunt

Unrest feels like

Dizziness, nausea

A storm that never stops

Trust that has been shattered

Unrest tastes bitter

Unrest sounds like a screech

Unrest is the sound of constant talking

When you cannot participate

This poem illuminates several aspects of poetic language as a resource, both as linguistic and creative expression and as a lyric manifestation in the group, the latter aspect underscoring how the poem established a dialogue by bringing the different voices of the group together. One of the participants exclaimed after the reading and discussion of the poem that it had created a *reflection room* in the group by opening up different ways of looking at emotions, of seeing how metaphors and similes could visualise feelings that were otherwise unacknowledged, unarticulated or mute. Another aspect of the poem was how the participants got glimpses into each other’s meaning making, which is highlighted by how one participant explained how the disturbing noises of people talking outside the meeting room ended up as the last stanza of the poem. The fact that this was a familiar sound in the group (as the hall room outside the room was an open space with a coffee machine) impressed the other participants in how the participant created an evocative metaphor of unrest out of the familiar. Furthermore, one of the other participants also found this stanza to be a vivid metaphor of psychosis. The poem highlights the reflexive aspects that became an integral part of the group. As observed during the sessions, and also expressed in the interviews, the participants acknowledged how the reading and comments from the group leaders and the other participants, made them see different aspects and qualities in their own and in others’ writing. It sparked a process where the participants learned more about creative writing.

The poem above also underscores how the creative process of writing, exchange and reflection in the group was connected to *playfulness*. At its best, the creative writing group was a room where they could play with words, expressions, images and literary techniques in short designated writing exercises. In particular, the writing exercises like the one above were liberating. They took little effort and time and promoted spontaneity. Other times, we wrote flash fiction (six word stories) and haiku poems and put them on the flip chart. Characteristically, these short exercises on the flip chart were there for all to see and discuss, creating a lot of *joy* and laughter in the group that was based on the sharing and improvisation but also in response to other literary examples.

### Writing as a ‘long table’

A prerequisite for the above category is the need for the participants to feel safe and comfortable in the group. This is highlighted in David’s text about the group, which was written at the last group meeting:

**The long table**

Testing, testing. Are you on, yes? Yes. Ok let’s roll. What do you think? No, wait, I can’t ask. Or can I. No. I can’t sit here and interview myself. And ask myself? How has it been? Well, I know fucking well myself that. It. Has ... wait, wait. Okay, I have got a good one here ... how do you feel? Well for me it has been a long table ... Long table? Well, in therapy there is a sofa and a chair and a fucking discreet notebook ... and an ink cartridge of a pen. On a course, there may be benches, but here it is a long table. Like in Harry Potter. Hogwarts. Narnia. What do you mean? I just mean that the reason I was able to write here and failed it at school was because ... there is no square box. Not a fucking war. We flee from the neglect of the family and go to [name of the hospital] and write ... Narnia / [hospital] / Hogwarts. And sorry Kristin, Hilde and Oddgeir. You are lovely. But ... our heads rule, we own this. But the course then? That’s the answer. The course proved. We all have something in common. We are alright together.

Several aspects of the importance of the group environment can be found in this text. The title ‘The Long Table’ alludes to the magic world of Harry Potter and how this implies a different logic than the sofa, chair and notebook in therapy sessions and the benches and the square box at school. In the interview, David explained how the writing group became a haven outside of the pressure of society. The importance of the group as a safe space was highlighted by all participants. In the case of David, he was very sensitive to pressure and expectations, being an ‘extreme perfectionist’, as he expressed. David needed to know that he could withdraw from writing exercises and bring other texts that he had prepared in advance instead if he did not manage to write something during the group session. However, because of this freedom, David experienced that he flourished as a writer: in the interview, he claimed that he had written more than ever because of the course. The metaphor of ‘the long table’ also implies equality among the participants themselves and among the participants and the group leaders. For David, it was crucial that the group was a *community of writers*. At the same time, he also stressed in the interview how critical it was that he was met as a fellow human being. Repeatedly during the group sessions, he underlined, ‘We are more than our diagnosis!’ In a similar vein, Nick expressed in the interview how he found the group to be a ‘sanctuary’ (‘fristed’) and stated, ‘I have felt free, and have felt more like a real person […] I have not thought of myself as a patient during these meetings. And that is something I immediately do when I walk out that door’. Joni expressed how she felt freer in the group; she experienced that there was space, also for the parts that normally do not fit. The group became a space where she could be calm and get some rest. In addition, Gordon pointed out that it felt liberating that the focus was on the texts and not on him and his situation. He underlined how he experienced that the group supported each other and that he had always looked forward to going there. Cindy said how the group had made her get to know the other participants, something that had not happened before, despite several of them being together for extended periods during treatment: ‘We didn’t have any common interest before we found one in the writing’. The experiences from the group, seen together with the participants’ expressions, underline how the writing group as a supportive and free space can release the potential for writing as well as (therapeutic) community.

The overall impression from the texts, the observations done in the group and in the interviews is that the group was experienced as a safe and inclusive space. At the same time, sitting together at the ‘long table’ is a fragile and precarious space that can easily be shattered or put at risk. At one of the meetings, a writing prompt was introduced that asked the participants to write about a particular smell. For one of the participants, this became a too difficult exercise because it brought forth traumatic memories, and the participant ended up leaving the room. This episode underscores how crucial it was to have a health professional attending. On this occasion, the psychiatrist went after the participant and talked about the experience. It ended with the participant coming back to the group after a short while, and the group and the leaders were able to have a conversation around what had happened. It was then decided to give another prompt that became ‘the easiest thing is …’. This episode was referred to in several of the interviews where the participants stressed how important it was that the group leaders be flexible and attentive to what happened in a potentially fragile group setting.

### Writing as personal discovery

Although the previous two sections have underscored the importance of the group setting as a supportive and creative space, this section emphasises how writing can involve a discovery of resources, thoughts and reflections in the writer. As Nick wrote in one of his texts:

I like to write. Writing gives me an overview of thoughts, as well as a way of expressing myself. I don't like to read. Unless I have written it myself. Writing is an art that takes time to learn and something you can never master. When you write, time stands still. Being part of the writing group has been a privilege. I feel that I get better when I have written for a while. Writing is a way of leaving something valuable.

Although Nick enjoyed being part of the group, he explained in the interview how he preferred to write alone and how he always had the writing prompts in the back of his head. Nick made lyrics before he started in the group, but he said that his writing had become better because he felt that the group had made it easier to write more freely. In the text above, Nick expresses how writing for him is a way of understanding his thoughts, an expression of himself and as representing a possible legacy. David explained that he had written more than ever after attending the writing group. (David had also written before the group). Although he sometimes struggled to write during the short, designated writing exercises in the group, he always brought texts into the group, and he explained in the interview how the writing helped him clear his thoughts. Gordon underlined how the writing both involved a learning of writing in different styles and a way of learning a bit more about himself, not least that he now dares to be a bit more open in his writing. As he elaborated in the interview: ‘Other things come to mind when I write: nice things, bad things, but not the least honest things. I am honest when I write. It’s a bit unfiltered somehow. I am honest when I talk too, certainly, but I believe I hold more back. That’s why writing is great’.

Another text that thematises writing as personal discovery is the following poem by Cindy:

Writing is for me

Like a song

I’ve never sung

Like a bee

I’ve never been stung by

Like a thread, I cannot sew

Writing makes me new

Cindy’s poem touches on several aspects of her writing that she also underlined in the interview. While the other participants had written before the group, Cindy said that she had learned to like to write, something she had never experienced before (like a song I’ve never sung) and something that also involved a discovery (writing makes me new). In the interview, Cindy elaborated on how she never had liked writing at school and that she had regarded herself as one who could not write. Now, she described writing as a way of ‘making sculptures with words’, and she found significance and depth in her language during creative writing. From first having negative experiences of writing, Cindy now found herself writing everywhere, at the bus or whenever she had time.

In the previous section (the long table), we saw how the group represented a ‘safe space’ and ‘sanctuary’ that might imply a therapeutic community. We would also like to highlight how writing as personal discovery might involve therapeutic aspects. Cindy claimed in the interview that her writing shifted the focus from thoughts of the illness to other aspects. Gordon reflected on how the writing might be therapeutic because he experienced that he had developed a broader understanding of the other group members as well of himself. In one of his texts on writing, he dwelled on how writing can involve a form of working through: ‘It is good to write, even if it sometimes involves bad things. It is anyway good to get it on paper, then everything becomes a little bit different’. David most clearly stressed that he saw writing as therapy and claimed that he has felt better through writing. David was also the one who most explicitly wrote about mental illness in several of his texts. However, not all participants thought writing was therapeutic. Nick, who in the quoted text above wrote, ‘I feel that I get better when I have written for a while’, stated in the interview that he did not think of the writing group as therapy: ‘It is something completely different’. Joni also claimed that the writing group was something else; it was not therapy but ‘a room within a room’, as she said in the interview.

### Writing as mystery

Writing as something ‘completely different’ or a ‘room within a room’ underlines how the writing group experience contained aspects that was not easy to pinpoint; there were elements that the participants had difficulties explaining but that were still a vital part of the writing experience. Cindy reflected on this in the interview when she pondered how writing ‘…might not reveal much, but can still affect the reader, through what it contains from the writer herself’. In addition, Nick hinted to the fact that writing may involve ‘…an unknown element… that is difficult to put your finger on’. In a similar vein Joni mentioned how the texts and their reception can hint to things that are not necessarily explicitly mentioned in the text: ‘For me it (the writing group) was like a white room that could contain multitudes. Also, things that are not easy to put in words’. A textual example by Joni can perhaps illuminate this, a poem that might or might not contain expressions of mental illness:

Do I hear or do I not hear

The voices do not exist

They are mirrors of the love

I did not receive from you

These are my twisted memories

That I cannot turn back

To the light

Another salient example of writing as mystery can be the ending of a poem Joni wrote in one of the last meeting to the writing prompt ‘What writing means for you’:

Falling leaves on paper

In my mercury mind

Words linger

‘Falling leaves on paper/In my mercury mind/ Words linger’. These lines might be a fitting example of this category, emphasising writing through metaphor that eludes elaboration. Another text is Gordon’s poem titled ‘The Writing Room’, where he ends the poem on what writing means for him with the following stanza:

These words

Therefore

How they are

And become

What they

Want to say

## Discussion: a poetics of vulnerability?

In the analysis, we asked *how poetic language can be a resource for young adults living with psychosis through participation in a safe and supportive creative writing group.* In the results, we have outlined how a selection of texts, together with extracts from the interviews from the participants, have underscored that poetic language can indeed be a resource for the participants. In the following, we highlight some of the above findings in light of our theoretical framework and previous research.

Ricoeur’s philosophy underscores the productive work of poetic language and metaphor (Ricoeur 2008b, Valdés 1991a) and how this might offer perspectives of how we understand and orient ourselves in the world. The practice of creative writing gave access to a poetic and productive language in the group and among the participants. One example is how the exercise of feelings and metaphor gave way to a reflexive room in the group, opening up nuanced understandings of language and the potential in the participants themselves and among the group members. The experiences of this exercise underscore the cocreative aspect of both the writing and the subsequent exchanging of perspective and reflection in the reception of the poem. The latter is accentuated by how the participants saw themselves and each other in a different light through the poem, underscoring Ricoeur’s point that poetic language can open for an understanding of the subject ‘in front of the text’ (1981) by proposing a world we can inhabit. In Ricoeur’s existential hermeneutics, this involves the possibility of an enlarged self through available creative discourses, something we believe hints at the experiences that became part of the group.

Furthermore, when Ricoeur argues that poetic images must be seen as *verbs*—as potential redescriptions of the world—we find it fruitful to see this in relation to other aspects of what an expressive, poetic language opened up for in the group. We saw how the participants found new resources in themselves through creative writing, like when Cindy discovered that she actually *could* write and that she liked it. In addition, a creative language opened up new ways of knowing oneself, as well as knowing others. As Cindy explained, she was surprised by how the writing group made the members get to know each other. Despite the fact that they had been together for longer periods during treatment, this was the first time they engaged with each other, realising that they had something in common. Another example is Gordon’s experiences of how things became different when he put them in writing, as well as learning more about himself and the others through writing and talking about the texts. Kristeva’s emphasis on poetic language as dialogic also underscores this central aspect of what we found in the texts and among the participants. A practice of creative writing potentially involves a dialogue with oneself and with the texts produced in the group, as well as among the members in the writing group.

We also look at how Kristeva’s argument that poetic language can counteract an oppressive symbolic order might point to aspects of the writing group. Here, we consider David’s words about how he found therapy to be ‘a sofa and a chair and a fucking discreet notebook’ held together with his need for being treated as more than his diagnosis. We can also look at Nick’s claim that he had felt more like a real person in the group. We believe that these experiences are closely related to how poetic language and the process of writing, as well as the reception of the texts, *do* something. They open a way out of a fixed understanding of the group members as patients with mental illness towards potentiality and agency. After one of the group sessions, the second author remarked on how the participants’ linguistic expressions were met with genuine curiosity and appreciation and were taken seriously by the group leaders and the other participants. Nothing written or expressed was deemed wrong or awkward. The experience of mastery in the practice of creative writing and reception hints at a different symbolic order than what the participants were used to in meetings with the health care system and maybe the school system before that. For instance, in the example where Nick expressed how he had never thought about the fact that he was a patient when he was part of the group.

Furthermore, Kristeva argues for how mental illness, when strengthened by the semiotic, can be a resource, a type of dark matter for creativity. All three authors of the current article were astonished by the creativity and linguistic originality that were present in the group, more than we have experienced in similar creative writing groups among cancer patients. David seemed to agree with this when he concluded in his text ‘The long table’:‘our heads rule, we own this’. MacCabe et al. (2018) has explored the association between studying a creative subject at high school or university and later mental disorder, finding that people studying artistic subjects at university are at higher increased risk of developing a severe mental disorder. MacCabe et al. (2018) point to two putative reasons for this: evolutionary genetics and psychological explanations. The first has regained attention because of a recent demonstration of an association between polygenic risk scores for schizophrenia and bipolar disorder with membership in artistic societies or creative professions (Power et al. 2015). The latter points to cognitive styles that may be associated with both creativity and psychosis, such as divergent thinking patterns (Folley and Park 2005; Gibson et al. 2009). If this is the case, creative writing and other forms of art therapy may be especially suited for people with severe mental health problems. This should be investigated further.

Another important finding from this work that we would like to reflect on is how poetic language opens up nuanced understandings of potentiality and agency through play and wonder. This is part of what Ricoeur (1976) terms the ‘surplus of meaning’, underscoring how poetic language can never be seen as a duplication of the world; instead, it must be seen as a metamorphosis. In particular, this is apparent in what we have highlighted under the findings as ‘writing as mystery’, an opening towards linguistic creative expressions that hint at depths that cannot be exhausted. The texts are open to the self and the world in a way that is genuinely poetic and productive, where both experiences from illness and other aspects of life can be put in writing without being dissected or defined.

Our experiences from this pilot underline that a creative writing group can be a valuable practice for young adults who have experienced psychosis. Although the research on creative writing in mental illness and psychosis is still in its infancy, our findings correspond with previous research that highlights creative writing as part of a recovery process in how it involves symbolisation and self-expression (see, e.g., Houlding and Holand 1988; Kar and Barreto 2018; Shafi 2010; Bundesen and Rosenbaum 2020). Researchers draw attention to how an emphasis on *literary technique and processes* opens for cognitive remediation (King et al. 2013) and an opening towards processing outside of the subject (Bundesen and Rosenbaum 2020). Moreover, the importance of the group as a place for positive affirmation and group affiliation is also found in our pilot and has been highlighted by others (e.g., Houlding and Holland 1988; King et al. 2013; Bundesen and Rosenbaum 2020).

A creative writing group might not be for everyone. It is essential that this stay a voluntary offer, but it should not preclude patients who have never written before or who are in different phases of treatment. We experienced that participants were in different phases, and we also saw them evolve and get better through the course. Our experience is that there was great support in the group, even though the participants were a mixture of in- and out-patients in the same group. Some had written before, and some had joined the course more out of boredom and were pleasantly surprised. In line with King et al. (2013) and Bundesen and Rosenbaum (2020), we stress the importance of literary competence in the arranging of creative writing groups. The importance of learning and listening to each other’s texts as literary expressions was vital for group development and the experiences of the group members. Furthermore, we would like to emphasise the need for flexibility in how the participants could be free to withdraw from writing assignments in the group or bring texts outside of the writing prompts.

### Closing reflections

We would like to end this discussion by once again highlighting Kristeva’s ‘ethics of linguistics’ (1984a) and Ricoeur’s ‘poetics of being’ (1950) as a contribution to understanding the potential of poetic language in mental health care. As we have seen in the writing group, the practice of creative writing can give affordances to subjects in movement and subjects in becoming in a safe group environment. As a creative community, and by being in dialogue with cultural and literary resources in the culture, poetic language can be part of a recovery process where the subject can find a voice, hope and connectedness. An opening towards creative language, as we have seen in the texts and in the expressions of our participants, can truly be poetic in the way that it creates, rediscovers and enchants the world. Thus, poetic language might open towards relatedness to oneself, to the group and to the world.

## Data Availability

Because of the written informed consent, the data are not available to other researchers.

## Footnotes

1 It must be noted that when Ricoeur is using the term ‘poetic language’, this is not only related to poetry but also to fictional narratives and also other creative linguistic genres such as personal narrations, which are influenced by fictional narratives.
